# Early Results of States’ Efforts to Support, Scale, and Sustain the National Diabetes Prevention Program

**DOI:** 10.5888/pcd14.170478

**Published:** 2017-12-07

**Authors:** Yvonne Mensa-Wilmot, Shelly-Ann Bowen, Stephanie Rutledge, Jennifer Murphy Morgan, Timethia Bonner, Kimberly Farris, Rachel Blacher, Gia Rutledge

**Affiliations:** 1 Division of Diabetes Translation, Centers for Disease Control and Prevention, Atlanta, Georgia; 2 ICF International, Atlanta, Georgia; 3 Oak Ridge Institute for Science and Education, Oak Ridge, Tennessee

## Abstract

The Centers for Disease Control and Prevention (CDC) developed a cooperative agreement with health departments in all 50 states and the District of Columbia to strengthen chronic disease prevention and management efforts through the implementation of evidence-based strategies, such as CDC’s National Diabetes Prevention Program. The National Diabetes Prevention Program supports organizations to deliver the year-long lifestyle change program that has been proven to prevent or delay the onset of type 2 diabetes among those at high risk. This article describes activities, barriers, and facilitators reported by funded states during the first 3 years (2013–2015) of a 5-year funding cycle.

## Introduction

Prediabetes is clinically known as the stage between normal blood glucose and severe glucose intolerance (1) where blood glucose or glycated hemoglobin A1C levels are elevated but not high enough to be diagnosed as diabetes. The Centers for Disease Control and Prevention (CDC) estimates that 84 million adults aged 18 years or older in the United States have prediabetes, nearly 90% of whom are unaware of their condition (2). Prediabetes increases the risk of developing not only type 2 diabetes but cardiovascular disease as well (3). The progression of prediabetes to type 2 diabetes can be prevented or delayed by lifestyle behavior modification addressing diet, exercise, and stress reduction that results in a 5% to 7% weight loss (3,4). On the basis of findings from the Diabetes Prevention Program research study and subsequent translation studies (5,6), Congress authorized CDC in 2010 to establish the National Diabetes Prevention Program (National DPP), which provides a framework for type 2 diabetes prevention efforts in the United States.

A key component of the National DPP is a structured, evidence-based, year-long lifestyle change program (LCP) to prevent or delay onset of type 2 diabetes in people with prediabetes or at risk of developing type 2 diabetes (7). The LCP is group-based program that is facilitated by a trained lifestyle coach, and uses a CDC-approved curriculum. The curriculum uses regular opportunities for direct interaction between the lifestyle coach and participants, builds peer support, and focuses on behavior modification through healthy eating, increasing physical activity, and managing stress. The program may be delivered in person, online, or through a combination of both delivery modes (8,9,10,).

CDC’s Division of Diabetes Translation works collaboratively to scale and sustain the National DPP through partnerships with public and private organizations at state and local levels (7). The Division of Diabetes Translation also manages the Diabetes Prevention Recognition Program (DPRP), the quality assurance arm of the National DPP. Through the DPRP, CDC awards recognition to organizations delivering the LCP that are able to meet national quality standards and achieve the outcomes proven to prevent or delay onset of type 2 diabetes (11). 

In 2013, in an effort to promote an integrated model of chronic disease prevention and management, CDC’s National Center for Chronic Disease Prevention and Health Promotion developed the State Public Health Actions to Prevent and Control Diabetes, Heart Disease, Obesity and Associated Risk Factors and to Promote School Health (SPHA-1305) cooperative agreement. Under this 5-year cooperative agreement, all 50 state health departments and the District of Columbia were funded to implement strategies to reinforce health promotion, epidemiology, and surveillance activities and implement targeted strategies that would have a significant impact on school health, nutrition, obesity, diabetes, heart disease, and stroke.

We present preliminary findings from a collaborative effort between CDC and state health departments designed to scale and sustain the National DPP. Findings from the first 3 years are described with the goal of providing an in-depth understanding of types of activities implemented along with barriers and facilitators experienced. Comments from grantee reports are included to augment findings presented. The information described in this article was exempt from ethical research approval because it involved only a secondary analysis of state program reports. Grantees did not report data in year 1; thus, findings reflect years 2 and 3 of the funding cycle (reports submitted in 2015 and 2016).

## Implementation Framework To Scale And Sustain the National DPP 

Through funding to state health departments, CDC works to promote awareness of prediabetes and the National DPP; increase prediabetes screening, testing, and referral; and increase program participation by facilitating relationships between government agencies, community-based organizations, insurance providers, private-sector employers, academia, and health care providers (8). State health departments also work to secure the program as a covered benefit for state employees and Medicaid beneficiaries at risk for type 2 diabetes.

The strategy for scaling and sustaining the National DPP is a set of recommended activities grouped into 4 drivers that are essential to long-term success: 1) support the efforts of partners to increase the availability of LCPs, 2) implement referral policies and mechanisms, 3) establish payers and payment mechanisms, and 4) identify and enroll people with prediabetes or at high risk for type 2 diabetes in LCPs (Table 1). When targeted individually and collectively, these drivers are designed to improve availability of programs; expand reimbursement and insurance coverage; increase the use of practices and policies within health care systems to screen, test, and refer patients; and increase willingness of people at high risk of developing type 2 diabetes to enroll.

**Table 1:** Abbreviated List of Activities from the National Diabetes Prevention Program Technical Assistance Guide

Driver	Activities
Support the efforts of partners to increase the availability of LCPs	Integrate LCP planning and implementation with ongoing state/city diabetes coalition activities or state diabetes action plans. Explain readiness criteria to organizations interested in becoming LCPs. Use grant funds to help ADA/AADE DSME programs develop a strategic business plan to determine their capacity to offer a LCP.
Implement referral policies and mechanisms	Distribute the AMA/CDC provider tool kit, and engage health care systems and providers in using it; partner with state and local medical associations in reaching the clinical community. Provide technical assistance, training, and academic detailing (face-to-face education of providers by trained health care professionals) on prediabetes screening, testing, and referrals to health care providers and care teams within existing LCP service areas. Support health care systems in building EHRs or other systems to facilitate and track referrals and enhance decision support.
Establish payers and payment mechanisms	Develop a state-specific business case for the National Diabetes Prevention Program. Work with state employee health plans and the state Medicaid agency to secure or extend coverage where needed. Encourage LCP providers to connect with third-party administrators where necessary to facilitate billing and reimbursement.
Identify and enroll people with prediabetes or at high risk for type 2 diabetes in LCPs	Use strategic communication strategies (eg, customized waiting room advertising) to reach people at high risk about the importance of National Diabetes Prevention Program benefits and coverage. Provide advanced training for lifestyle coaches (eg, motivational interviewing) to further strengthen group facilitation skills. Provide materials and other resources to support existing LCP providers’ marketing efforts to recruit participants.

Abbreviations: AADE, American Association of Diabetes Educators; ADA, American Diabetes Association; AMA, American Medical Association; CDC, Centers for Disease Control and Prevention; DSME, diabetes self-management education; EHR, electronic health record; LCP, lifestyle change program.

## State Health Department Progress on Key Activities

State health departments provide an account of their progress for key activities and outcomes to CDC annually. Two CDC authors (Y.M. and S.R.) qualitatively analyzed data from grantee annual performance reports from years 2 and 3 to summarize the types of activities implemented. We developed structural codes from the drivers and analyzed 20 activities from each data set to ensure reliability of the codes. To ensure consistency in coding, authors then discussed their findings and refined codes to reach a consensus before independently analyzing the remaining data. During their discussion, authors added codes to capture activities that were inconsistent with the drivers. Activities that were dropped by state health departments were not included in the analysis.

### Barriers and facilitators encountered by state health departments

State health departments selected 1 intervention strategy to evaluate over the cooperative agreement period. In year 2, California, Colorado, Florida, Kentucky, Maine, Maryland, Minnesota, Missouri, Montana, New Mexico, Nebraska, New York, North Carolina, Oregon, and Rhode Island chose to evaluate the National DPP strategy. In year 3, Nebraska elected not to evaluate its National DPP work. Evaluation reports from 15 states were included in the year 2 analysis, and reports from 14 states were included in year 3. Two authors (S.B. and Y.M.) analyzed the 2 data sets by using a multistage iterative process to develop a hierarchy of codes for the data. Authors determined a priori to use the drivers as basic codes, then conducted a thematic analysis coding of reported facilitators and barriers to each driver. After an initial analysis of 2 reports, authors compared findings and refined and added subcodes before proceeding to code the remaining reports. An additional “other” category was added to capture information not classified within the codes and subcodes.

The number of activities implemented by state health departments across all 4 categories of drivers doubled from year 2 to year 3, from 148 to 295. State health departments engaged partners to support the scaling of the National DPP gradually and strategically through the funding cycle. A summary of key facilitators and barriers with representative comments is presented (Table 2).

**Table 2:** Facilitators and Barriers to Implementing the National Diabetes Prevention Program, 2015–2016

Themes	Comments From State Health Department Representatives
**Facilitators**	
Reimbursement availability	“State employees began having the National DPP lifestyle change program offered to them as a covered benefit. Our diabetes program has been working with the State Employee Group Insurance Program to promote the ‘Prevent’ program within our agency.” (Minnesota) “A large employer and a large insurance company announced (2017) that the National DPP will become a covered benefit. Expansion in insurance coverage is due in part to California’s Department of Public Health’s PDSTAT statewide organization of stakeholders, which has been instrumental in educating payers and insurance companies about the need for and value of the National DPP. The US Preventive Services Task Force recommendations on diabetes screening, released in October 2015, were another factor in encouraging adoption of coverage for the National DPP.” (California)
Practice/provider referral policies	“Based on CDC DPRP data, over 75% of participants in lifestyle change programs have enrolled based on a blood-based diagnostic test, which indicates that the majority of participants had a clinical test indicating prediabetes and were likely referred by a health care provider. YMCAs that established referral policies with local hospitals or health care providers show greater success in recruiting and filling classes than those that did not.” (New York State)
Program curriculum	“Having standard curricula and referral policies helps facilitate dissemination of the National DPP lifestyle change program in community settings, particularly since coordinated care organizations want to implement evidence-based programs.” (Oregon)
**Barriers**	
CDC recognition process	“Paperwork and complicated processes, as well as the inability to use grant funds to support direct services, have been a challenge.” (Maryland)
Limited program resources	“Several health systems, clinics, and community-based organizations are linked to lifestyle change programs for delivery and referral. However, many do not have formal policies and bidirectional networks in place. Staff and funding aimed at enhancing these policies and networks have been essential to carry this work forward.” (Nebraska) “These were the barriers to optimal National DPP implementation. There is a limited amount of wellness funding that has to be stretched across different priority areas.” (Colorado)
Reimbursement availability	There is no standardized method of reimbursement, and confusion exists about who within the health system can apply for reimbursement: “Lack of insurance coverage for the program often shuts down conversations about referrals and is a constant barrier. Despite these obstacles, we do have some early adopters who are developing policies or willing to undergo practice change.” (Minnesota)
Obtaining referrals	“Many lifestyle change programs report low enrollment and almost no referrals from physicians, even in cases where outreach was conducted to provider offices and larger health systems.” (California)
Participant cost	“Lack of insurance coverage for lifestyle change programs statewide is most often cited as a reason for why providers are not diagnosing and referring patients and why patients are not attending (due to the high cost of the program). There are only a small handful of insurers in New York State that are covering the National DPP as a benefit for their members.” (New York State)
Lack of data	“There is a lack of data on program completion rates, insurance information of enrollees, and measured health outcomes of program completers. Some insurers are aware of the benefit of the program but need more information on completers and outcomes to consider reimbursement.” (Rhode Island)
Lack of awareness	“The majority of employees were not aware of the health and wellness policies in place in their departments.” (Colorado)

Abbreviations: CDC, Centers for Disease Control and Prevention; DPRP, Diabetes Prevention Recognition Program; National DPP, National Diabetes Prevention Program; PDSTAT, Prevent Diabetes Screen Test Act Today.

### Support the efforts of partners to increase availability of LCPs

Thirty-five state health departments supported the implementation of activities of their partners to increase availability of programs in year 3, an increase of 133% from 15 state health departments in year 2 (Figure). The most commonly reported activities for this driver were creating a network of partners to develop a strategic plan to scale and sustain the National DPP, convening key stakeholders to address barriers affecting programs, examining state data to prioritize the location of new programs, establishing mechanisms to increase the availability of LCPs, identifying organizations with infrastructure and capacity to deliver programs, and leveraging state resources to support their partners to start new programs. State health departments partnered with community organizations, health care organizations, employers, private insurers, and government agencies to increase the availability of LCPs in the community.

**Figure:**
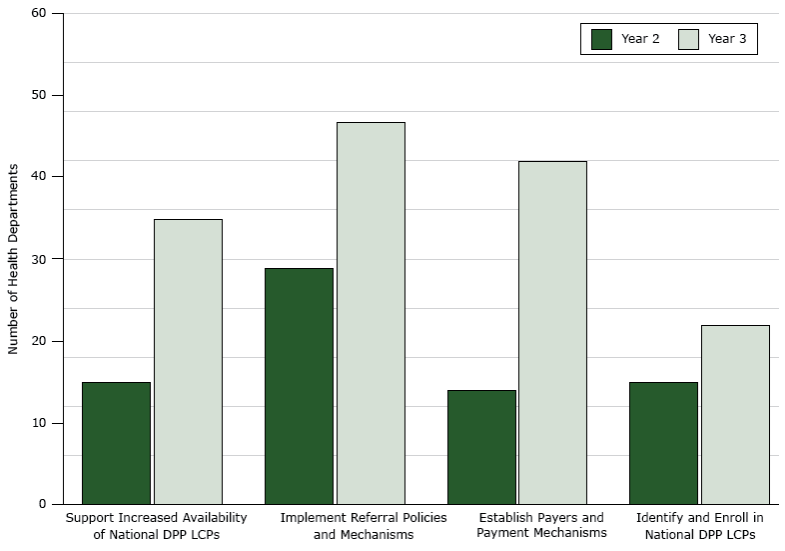
Number of state and District of Columbia health departments (n = 51) implementing activities within each of 4 drivers essential to increasing enrollment of people with prediabetes or at high risk of developing type 2 diabetes into National Diabetes Prevention Program (National DPP) lifestyle change programs (LCPs), 2015–2016. **Table:** 

Drivers	Support Partners to Increase Availability of National DPP LCPs	Implement Referral Policies and Mechanisms	Establish Payers and Payment Mechanisms	Identify and Enroll in National DPP LCPs
Year 2, 2015	15	29	14	15
Year 3, 2016	35	47	42	22

California reported “a marked increase in the number of in-person and online LCPs due, in part, to organizations and businesses that were able to host LCPs in multiple locations.” The ability of these organizations and businesses to obtain funding was reported as another facilitator that “removed key barriers to the start-up of new programs” (Minnesota) and “supported program uptake” (Maryland). Nebraska reported that “The complex nature of evidence-based programs made timely, clear, and adequate technical assistance important to our programs and enabled us to continue with our implementation efforts.”

Although partnerships were vital, some state health departments faced several challenges in their partnerships, including a lack of accountability, predetermined reporting structure, clarity in partnership roles, follow-through with strategic planning efforts, decision-making power, clear partner priorities, and interest. All of these challenges impeded efforts to increase program uptake. 

### Implement referral policies and mechanisms

Forty-seven state health departments implemented activities to increase the number of provider referrals made to LCPs in year 3. This represented a 62% increase in number of state health departments from year 2 (Figure). Of the 11,385 participants enrolled in LCPs in year 3, 67.6% (7,700) were referred by a provider. The most common activities reported were promoting the adoption of the American Medical Association (AMA)/CDC provider tool kit (12), providing technical assistance on prediabetes screening and testing, integrating referrals into coordinated care models, and leveraging existing electronic health records (EHRs) as novel referral methods to increase participation in LCPs partnering with state and local medical and nonmedical associations to engage the clinical community. For example, in years 2 and 3, the Florida Department of Health, in partnership with the American Diabetes Association (ADA), provided mini-grants to 14 LCPs. These grantees were able to reach 503 health care practices and 955 physicians to discuss establishing processes or policies for referrals to LCPs, and 33 policies and 256 procedures were implemented to refer patients to LCPs. In the 14 LCPs, 336 participants achieved the desired weight loss outcome of 5% or more in year 3.

Facilitators to developing referral policies for LCPs were having the buy-in of hospital systems, partnering with providers to establish patient referral policies, delivering provider education through academic detailing (face-to-face education of providers by trained health care professionals), providing feedback to providers on referral status, and integrating or linking CDC-recognized lifestyle change programs to referring clinics. Having LCPs attached to primary health care settings was valuable. For example, “YMCAs that established referral policies with local hospitals or health care providers show greater success recruiting and filling workshop classes than those that did not” (New York State). Integration of prediabetes clinical measures into EHRs and providing prediabetes resources in patient waiting areas contributed to referral success. Lifestyle coaches and participants viewed health care providers and workplace health programs as effective referral mechanisms. Grantees also reported challenges to increasing referrals, such as low provider awareness, provider resistance to making referrals, and difficulty reaching providers to establish a feedback loop.

### Establish payers and payment mechanisms

Activities to establish coverage through payers and payment mechanisms included convening stakeholders to develop a state-specific business case, recruiting champions, and engaging stakeholders to discuss coverage for state employees and Medicaid beneficiaries. By year 3, 42 state health departments were implementing activities around this driver, a threefold increase from 14 state health departments in year 2.

Establishing partnerships to address lack of coverage was key to increasing LCP reimbursement and enrollment for state health departments: “State employees began having the National DPP offered to them as a covered benefit. Our diabetes program has been working [for a while] with the State Employee Group Insurance Program to promote the ‘Prevent’ program within our agency” (Minnesota); “The National DPP was added as a covered health benefit for state employees enrolled in Kaiser Permanente and United Healthcare plans” (Colorado).

Lack of insurance coverage for the National DPP was reported as a significant barrier. One state health department expressed concern that the lack of insurance coverage for the National DPP transferred the implementation costs to delivery sites that depended on reimbursement to be sustainable. In this state, low or no availability of coverage is reportedly driven by a complex payer landscape where Medicaid reimburses for the National DPP, but not all employers offer insurance coverage for prediabetes. Another state health department established a formal relationship with the state governor’s office, which resulted in coverage for state employees. The governor subsequently established National DPP enrollment as a leading health metric for the state.

### Identify and enroll people with prediabetes or at high risk for type 2 diabetes in LCPs

In year 3, 22 state health departments were involved in activities to increase enrollment of people at high risk of developing type 2 diabetes into LCPs (Figure). This was a 46% increase in the number of state health departments implementing activities to support this driver from year 2 to year 3. The most commonly reported activities were training lifestyle coaches, developing a marketing plan, and directing culturally appropriate marketing materials to people at high risk of developing type 2 diabetes.

State health departments reported that availability of culturally and linguistically aligned lifestyle coaches was a major facilitator for identification and enrollment of people with prediabetes or at high risk for type 2 diabetes into LCPs. Transportation, proximity to programs, awareness of programs, maintaining contact with program participants on a regular basis, and availability of low-cost or no-cost programs were also reported as facilitators to increasing enrollment. The Montana state health department reported increased enrollment and participation in the LCPs and concluded that incentives contributed significantly to participants’ weight loss outcomes. Barriers to participants’ willingness to enroll were transportation needs, cost, scheduling difficulties, nonadherence to care, LCP complexity and length, lack of perceived self-efficacy, lack of skills needed to track food intake and physical activity, and feelings of discomfort in group settings. Another state health department reported that “the challenge continues in assuring that both consumers and clinicians recognize that prediabetes is a considerable risk factor and one that can be reduced with participation in evidence-based programming” (Nebraska). Solutions reported were using Medicaid transportation assistance, adapting the curriculum without changing core elements, reiterating key session points, simplifying tracking tools, promoting coping skills, and providing ongoing support from multiple people beyond the coaches (case managers, doctors, therapists, family, and friends).

### Limitations of this early analysis

Reporting from state health departments on their implementation of activities to scale and sustain the National DPP had some limitations. Because the evaluation of activities implemented to scale and sustain the National DPP was optional, only 15 state health departments in year 2 and 14 in year 3 elected to evaluate the impact of their activities. Thus, the discussion of barriers and facilitators to implementation represents what was reported by state health departments that evaluated their National DPP work. If state health departments opted to evaluate a strategy on the basis of how well they were doing, the overall results would appear more favorable than what actually took place. In addition, the variation in the level of detail provided in the annual performance reports and evaluation reports is a limitation of this study. Some grantee reports provided detailed accounts of activities, successes, and barriers, whereas other reports provided brief responses.

Despite aforementioned limitations, this report reflects efforts to promote an integrated model of chronic disease prevention and provides insights into ways to evaluate activities to support and scale a complex, multisector national program designed to stem the current and projected growth in new cases of type 2 diabetes. Our findings identify unique and innovative approaches for real-world program adoption and implementation — specifically, approaches that inform new ways of encouraging people in various sectors to work together to improve health. Our findings provide real-time insight that can be used to refine universal program implementation and increase opportunities for people at risk to be exposed to evidence-based interventions and to have good health outcomes.

## Implications for the Future

State health departments are effectively supporting evidence-based programs such as the National DPP to prevent or delay the onset of type 2 diabetes in people at high risk. Improving and sustaining collaborations between public health agencies and health systems is crucial to the success of this work. We present information on what works and also information for developing guidance on implementing activities that support and scale this evidence-based intervention in community settings. Understanding the activities being implemented, along with barriers and facilitators, has implications for technical assistance to support the expansion and sustainability of the National DPP. These results provide relevant data on state health departments’ progress and contribute to the identification of potential best practices. Furthermore, what is learned from states’ evaluations is critical to making adjustments midstream during implementation of activities to scale and sustain the National DPP. These early findings can inform the establishment of communities of practice, identify state health departments to lead peer-to-peer learning collaboratives, and shape guidance for scaling not just the National DPP but future public health practice.
